# Acknowledging our long-serving Associate Scientific Editors

**DOI:** 10.24095/hpcdp.45.9.06

**Published:** 2025-09

In 2025, *Health Promotion and Chronic Disease Prevention in Canada* (the HPCDP Journal) renewed its roster of Associate Scientific Editors. In that process we were lucky to gain new and talented Associate Scientific Editors, but unfortunately, a few long-serving editors have moved on.

***Margaret de Groh***

Margaret de Groh retired from the public service in 2024. During her long and exceptional career with Health Canada and the Public Health Agency of Canada (PHAC), Margaret was part of the inaugural group that forged a path for PHAC in 2004. Throughout her 20-year career at PHAC, Margaret played central roles in the advancement of scientific work on diabetes prevention (notably, the CANRISK project), nutrition and food security projects, and alcohol control policies and PHAC’s collaboration with the COMPASS and CLSA research platforms. 

Margaret was pivotal at the HPCDP Journal, with unwavering service and leadership from 2015 until her retirement. Throughout her tenure she held numerous roles, including Editor-in-Chief from 2017 to 2019, Associate Scientific Editor (ASE), subject matter expert—including on sex- and gender-based analysis—and Associate Editor-in-Chief. Regardless of her role, she consistently embodied kindness and prioritized individuals. Despite her busy schedule, Margaret was exceptionally generous with her time, always willing to listen, offer assistance and provide support. For many members of the HPCDP Journal team, Margaret was a mentor and an inspiration. 

Margaret's dedication and contributions have left an enduring mark on the HPCDP Journal. We extend our sincere gratitude for her years of service and wish her all the best in her retirement.

***Scott Leatherdale***

The HPCDP Journal acknowledges the significant contributions of Scott Leatherdale, who served as an ASE from 2017 to 2024. Before joining the Journal, Scott was a recipient of a CIHR-PHAC Applied Public Health Chair and had already established himself as a vocal and dedicated advocate for the Journal’s mission and its inherent value in the broader public health landscape in Canada. 

Scott is known by the Journal team as a steadfast and grounded individual, someone who always upholds his values and beliefs. He consistently demonstrated his keen eye for detail during his numerous thorough manuscript assessments.

The HPCDP Journal team extends its sincere appreciation to Scott for his years of dedicated service and wishes him well in his future endeavours. 

***Paul Villeneuve***

Paul Villeneuve served as an ASE from 2015 to 2025. During his decade of service, Paul was an invaluable member of the Journal team. He was routinely consulted for his tremendous expertise in biostatistics and his insights into the scientific landscape. 

Paul consistently demonstrated exceptional reliability in his timely manuscript assessments and communications. A dependable and efficient contributor to the editorial process, Paul was always willing to take on the most difficult editorial tasks, ensuring his standing as an integral team member. 

The HPCDP Journal team expresses its sincere gratitude to Paul for his decade of dedicated service. We wish him all the best in his future endeavours.

